# 3D modeling of the frontal sinus as a contribution tool for human identification - a case report

**DOI:** 10.1007/s00414-026-03815-z

**Published:** 2026-04-27

**Authors:** Sandra Gabolli, Ana Clara Oliveira, Paulo Miamoto, Maria Isabel Villalobos

**Affiliations:** 1 Present Address: Department of Medicine, Pontifícia Universidade Católica de Minas Gerais, Betim, Brazil; 2 Present Address: Department of Dentistry, Pontifícia Universidade Católica de Minas Gerais, Belo Horizonte, Brazil; 3 Forensic Odontology and Anthropology Section, Scientific Police of Santa Catarina, Florianópolis, Brazil; 4 Pontifícia Universidade Católica de Minas Gerais, Belo Horizonte, Brazil

**Keywords:** Forensic anthropology, Imaging, Three-dimensional, Forensic dentistry, Frontal sinus, Subtraction technique

## Abstract

Forensic identification in advanced decomposition body is challenging when traditional methods become unreliable. This case report demonstrates the utility of 3D modeling of the frontal sinus for human identification in forensic anthropology and odontology. A decomposed body found in southern Brazil was successfully identified using 3D-2D superimposition technique, which overlays a three-dimensional model derived from antemortem computed tomography (CT) data onto postmortem radiographs. The frontal sinus, with its unique morphological patterns, served as a reliable anatomical marker for comparison. This cost-effective approach utilizing open-source software addresses the need for accessible identification methods in resource-limited forensic settings. The technique demonstrates superior accuracy compared to traditional 2D radiographic methods while remaining practical for institutes with limited imaging technology. Our findings supporteas the application of 3D-2D superimposition and highlight the importance of forensic experts training in 3D forensic imaging technology for enhanced human identification capabilities.

**Supplementary Information:**

The online version contains supplementary material available at 10.1007/s00414-026-03815-z.

## Introduction

Human identification in individuals with advanced stages of decomposition constitutes a significant challenge in forensic medicine, particularly when traditional methods such as friction ridge analysis or DNA profiling become unfeasible [1–3]. In these cases, forensic odontology plays a fundamental role, primarily due to the individuality inherent in the structures of the maxillofacial complex, which can enable human identification. Technical and scientific expertise regarding the anatomical features of the face and dental arches is essential for the effective application of these methods, particularly in cases involving charred, skeletonized, or severely compromised soft tissues [3, 4].

Recognized as one of the primary methods of human identification - according to the DVI Guide [5], which also includes friction ridge analysis, DNA profiling, and surgical implants that may be unique to the victim - forensic odontology is usually associated with the use of teeth for identification. The use of maxillofacial regions is commonly performed by forensic anthropologists and should be well known by forensic experts dedicated to human identification, such as forensic odontologists. This knowledge should include the examination of the paranasal sinuses. Specifically, the frontal sinus exhibits unique morphological patterns in each individual, which can be compared in antemortem (AM) and postmortem (PM) examinations. Initially, such analyses were limited to two-dimensional (2D) radiographs; however, assessment of the frontal sinus has advanced significantly with the advent of computed tomography (CT) and three-dimensional (3D) modeling, thereby enhancing the accuracy and reliability of forensic comparisons [6–11].

Computed tomography (CT) has been widely incorporated into both dental and forensic practice, allowing for detailed anatomical reconstructions from multiple cross-sectional image [12]. When combined with 3D superimposition techniques, this approach enables a detailed and comprehensive assessment of individualized anatomy, while reducing artifacts and optimizing image processing time [13]. The scientific literature indicates that this methodology increases the accuracy of the identification process, establishing it as an indispensable tool in forensic institutes.

The present study aims to report a case that illustrates the practical application of CT and 3D superimposition in the identification of an individual at an advanced stage of decomposition, highlighting the potential of these tools in the routine practice of forensic anthropology and odontology.

## Case report

A body at an advanced stage of decomposition was found and transported to the Forensic Medicine Department (SML) of a city in southern Brazil. Given the impossibility of identification through other methods, the assistance of the forensic odontology service was requested to perform the necessary examinations. Following police inquiries, the family of the presumed victim was located and contacted. The family provided a CT scan with access to the original DICOM files, which was used to support the forensic odontological comparison. The forensic odontology team received only the cranial segment of the body, which was in an advanced state of decomposition, including skeletalized portions, areas of liquefaction, and a sectioned cranial vault for the examination of the cranial cavity.

**Fig. 1:**
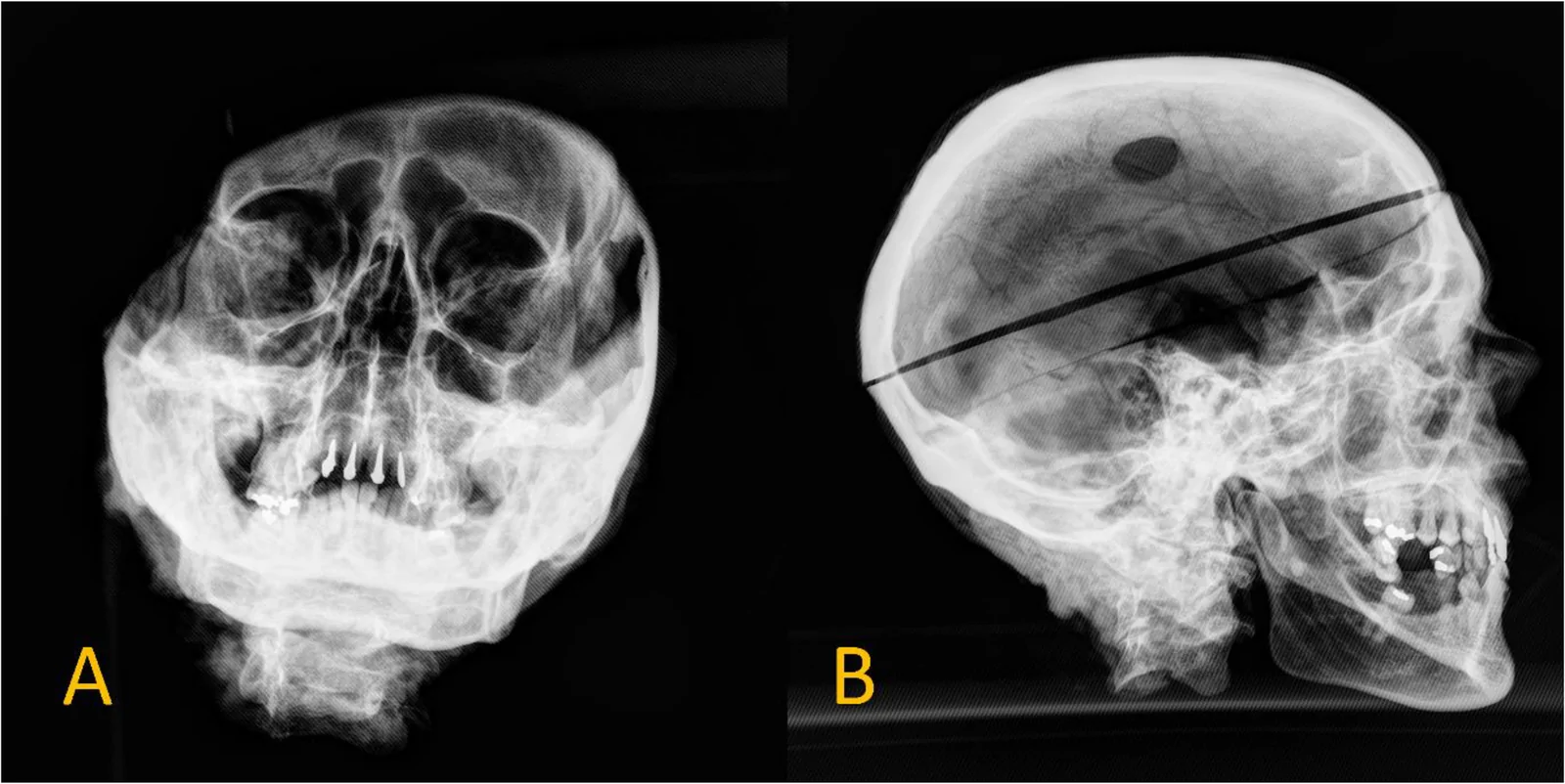
Radiographs of the paranasal sinuses: (**A**) frontal view and (**B**) lateral view

**Fig. 2:**
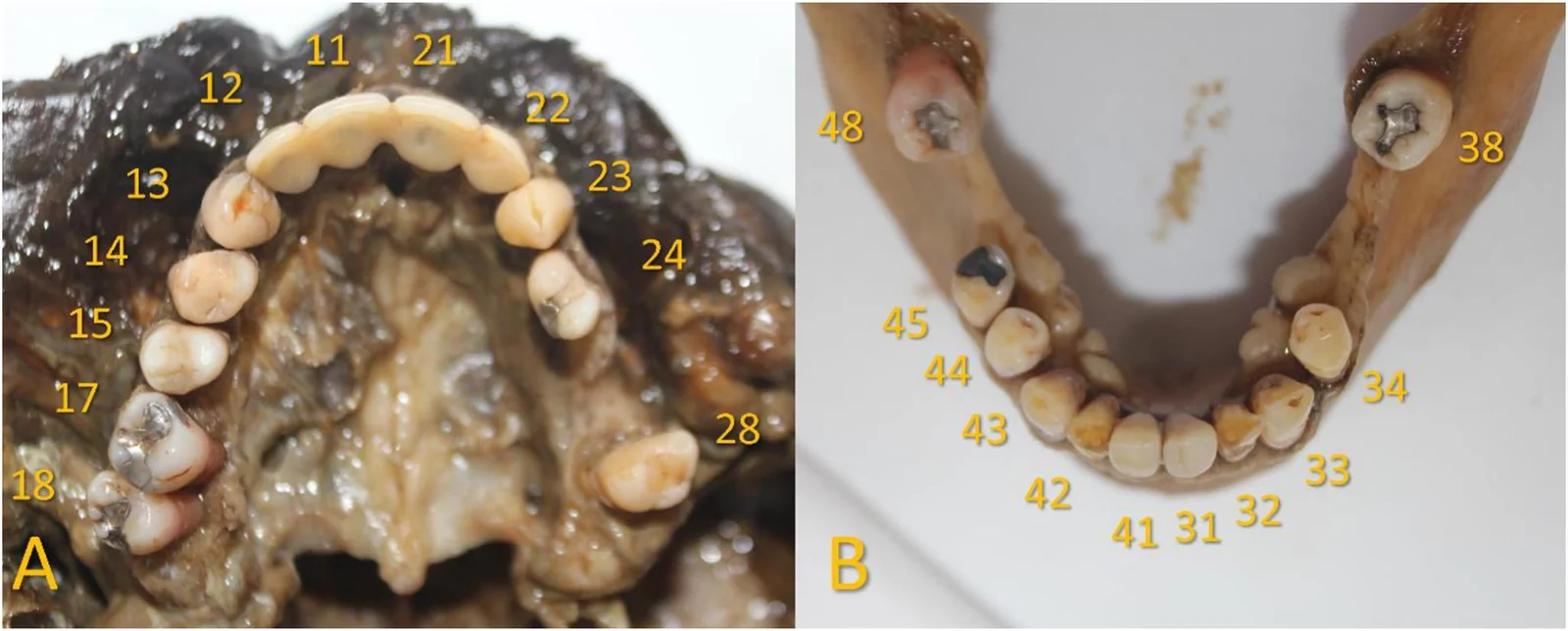
Maxillary (**A**) and mandibular (**B**) dental arches in occlusal view, with individual teeth identified according to FDI dental notation

The cranial segment was subjected to radiological examination of the paranasal sinuses to assess the remaining bony structures (Fig. 1). Subsequently, the mandible was removed to facilitate a detailed forensic odontological examination (Fig. 2).

A comparison between postmortem and antemortem examinations was performed, and no unexplained discrepancies were observed, with multiple positive matches recorded.

**Fig. 3:**
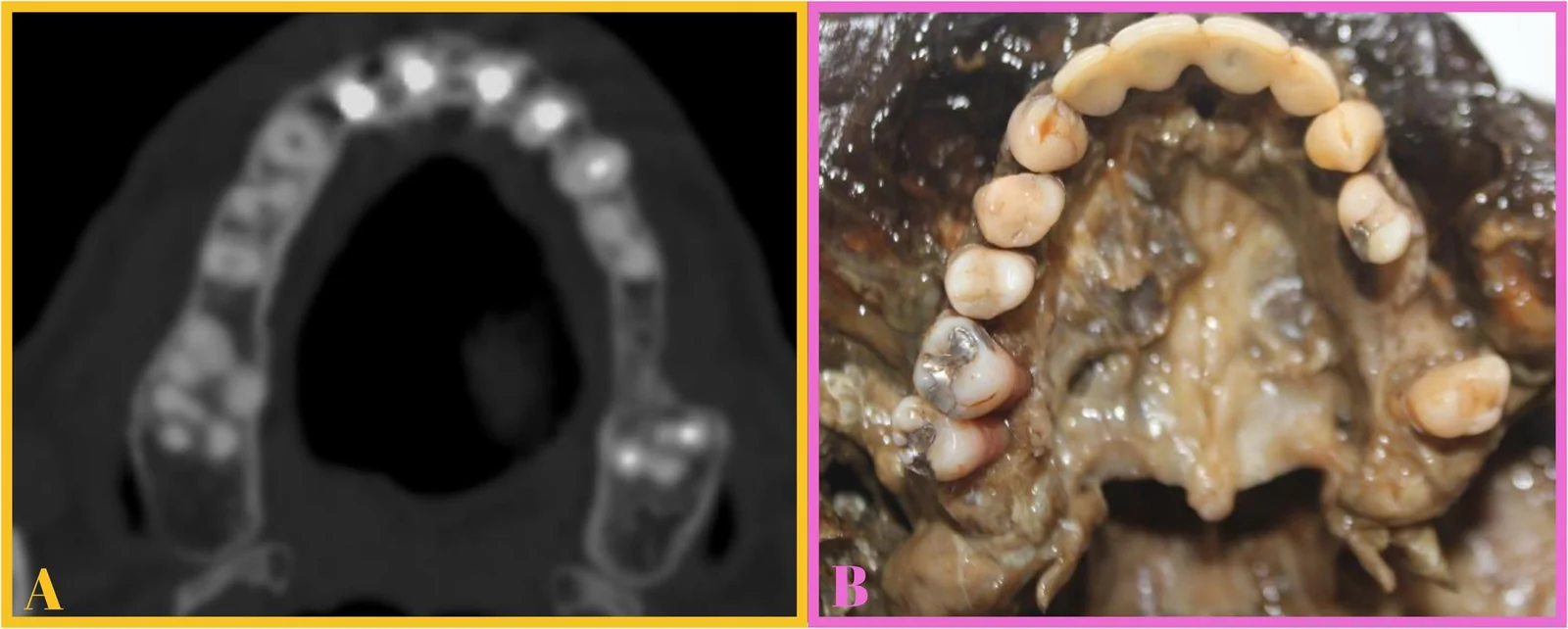
Forensic odontological comparison between an axial CT slice of the maxilla and the occlusal view of the maxillary arch, showing morphological compatibility as well as correspondence between present and absent teeth, their rotational position, and the presence of prostheses on anterior teeth

**Fig. 4:**
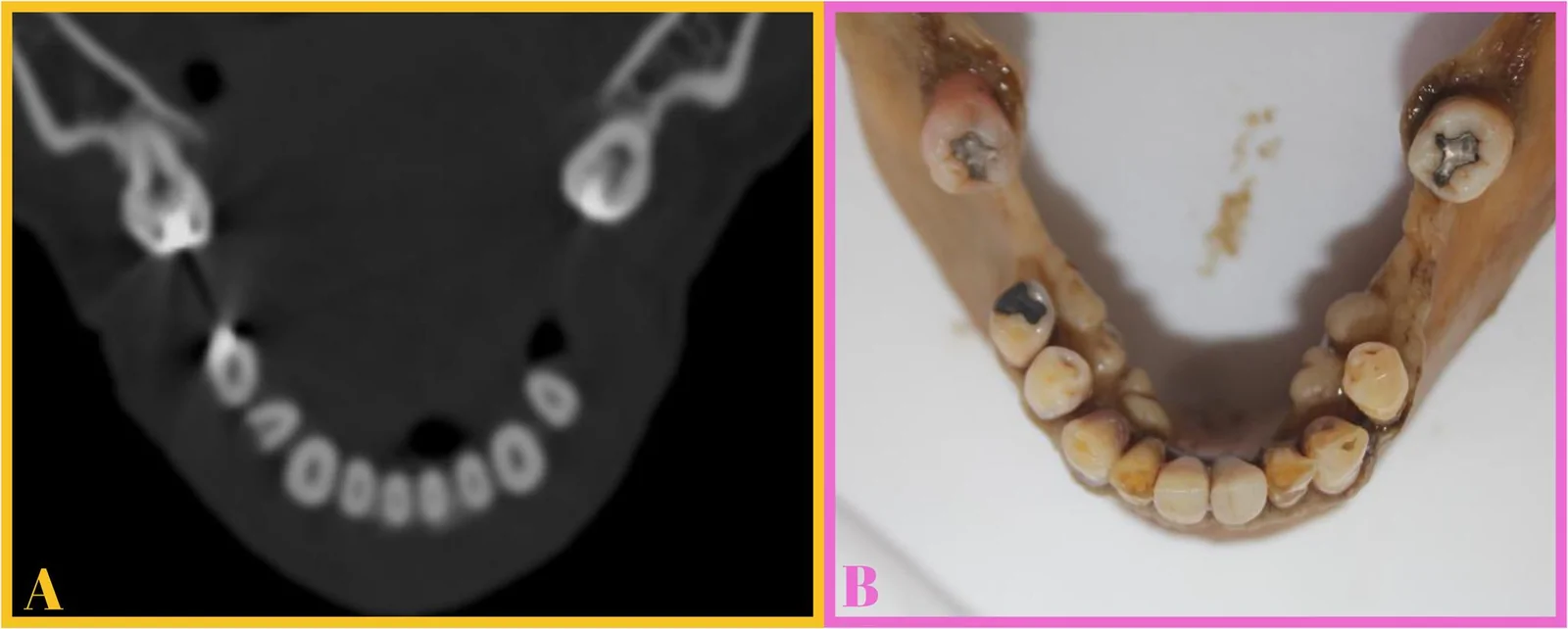
Forensic odontological comparison between an axial CT slice of the mandible and the occlusal view of the mandibular arch, showing morphological compatibility as well as correspondence between present and absent teeth, their rotational position, and the presence of silver amalgam restorations.

**Fig. 5:**
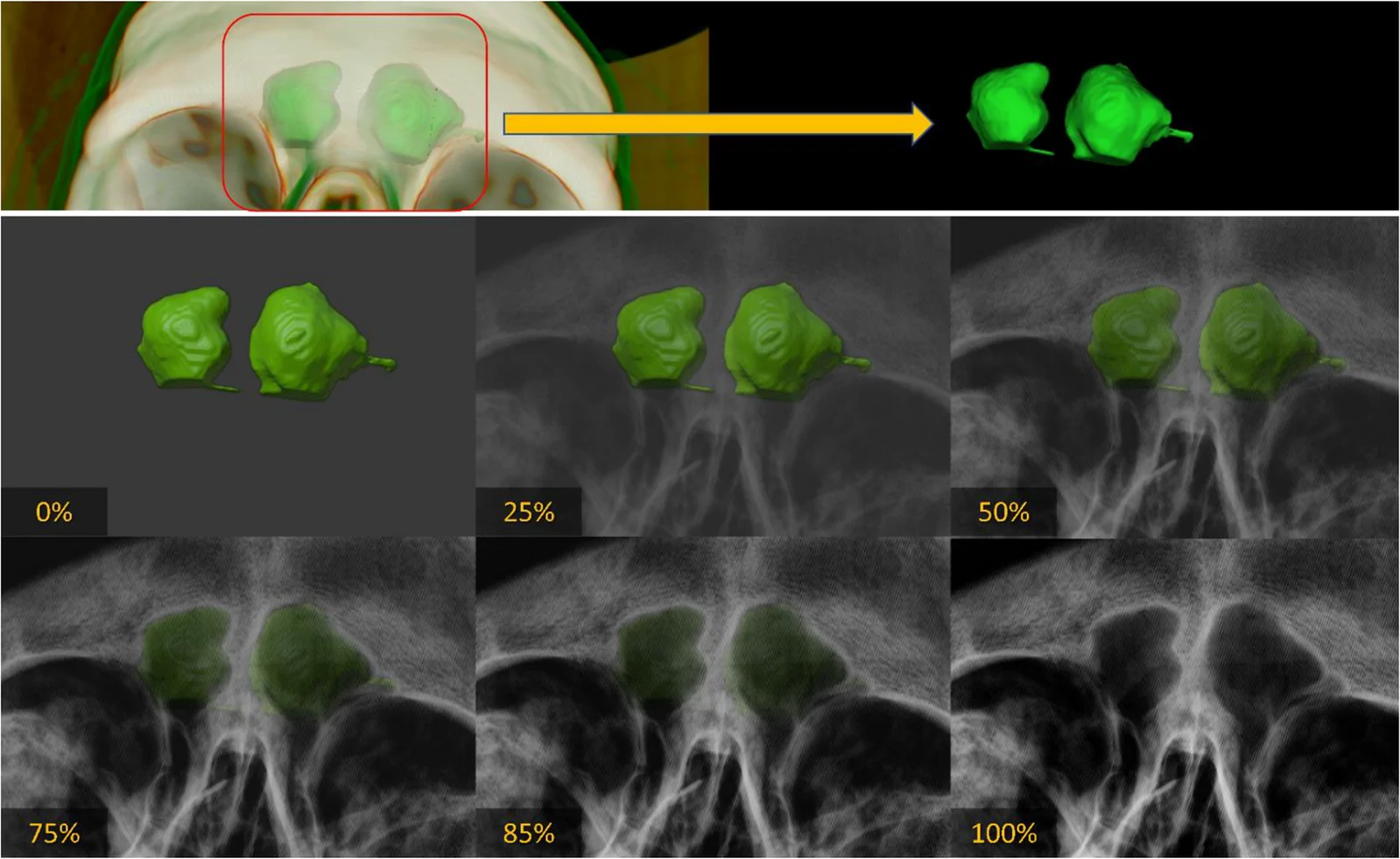
Process generating a digital 3D model of the frontal sinus from the reference CT. Below, this file was adjusted for scale and rotation on the PM radiograph, demonstrating morphological compatibility between the two records. The degree of radiographic image opacity is indicated in each segment

The morphology of the frontal sinus in the AM and PM examinations was analyzed, allowing for a detailed comparative assessment. To demonstrate morphological compatibility between the imaging of the missing person and the victim, direct comparisons were performed (Figs. 3 and 4), as well as the superimposition of a virtual 3D model onto a two-dimensional (2D) image. For the superimposition, a digital model of the frontal sinus was generated from the CT scan provided as antemortem material. This file was then imported into a virtual 3D environment, where it was adjusted in scale and rotation to align with the postmortem radiographic image, using the width of the frontal sinuses as a reference. Compatibility between the analyzed data was confirmed by the correspondence of anatomical structures in the anterior view (Fig. 5). 

## Discussion

The frontal sinus is widely recognized in forensic literature for its morphological uniqueness among individuals, which makes it a valuable anatomical structure for human identification. Located in the frontal bone and developed through cranial pneumatization, this sinus exhibits significant variation in shape, thickness, size, and symmetry [14–17]. These individual characteristics allow its use in human identification [6–8, 18–20].Traditionally, frontal sinus analysis has been carried out through two-dimensional (2D) radiographs or axial slices from computed tomography (CT), despite inherent limitations regarding morphological fidelity and superimposition of adjacent structures [9–11]. In the present case, comparative examinations using 2D radiography and CT were performed. These proved decisive for the application of AM and PM 3D modeling techniques, enabling detailed analysis of the frontal sinus and direct comparison with postmortem findings.

With technological advances, especially in imaging sciences, 3D methods have been progressively incorporated into forensic practice [6, 12, 13, 21, 22]. Computed tomography, particularly in its cone-beam modality (CBCT), enables accurate volumetric reconstructions of the skull, including the paranasal sinuses. This 3D visualization significantly improves anatomical interpretation and reduces errors derived from planar projections. In addition, 3D models can be stored, digitally manipulated, and quantitatively analyzed, which facilitates standardization of procedures and validation of findings [12, 13, 23].

3D–3D superimposition techniques have gained prominence as robust tools for human identification. By allowing quantification of congruence between two anatomical models, this approach offers a higher degree of accuracy compared with 2D comparisons or even mixed superimpositions [10, 24]. Studies such as Beaini et al. (2015) have demonstrated the applicability of 3D–3D comparison in frontal sinus analysis, reporting promising results in terms of accuracy and reproducibility [25]. Recent systematic reviews support the effectiveness of this method, even in structures that are difficult to standardize, including palatal rugae and sphenoid sinuses [21, 24–27]. In the Brazilian forensic context, where high-end imaging technologies such as CT are not universally available in all Forensic Institutes, methodological innovation becomes crucial to ensure the effectiveness and accuracy of human identification processes [28–34]. This case illustrates a promising development in this scenario by successfully applying a 3D–2D superimposition approach. Although methodologically challenging, it opens new possibilities for IMLs with more limited infrastructure. The 3D–2D superimposition technique was successfully applied to compare AM and PM models of the frontal sinus, enabling detailed evaluation of morphological compatibility between structures and contributing significantly to the identification outcome, thus confirming the method’s effectiveness.

Traditionally, forensic identification based on imaging has evolved from two-dimensional (2D–2D) analyses, as exemplified by conventional radiographic comparison, to three-dimensional (3D–3D) methods using CT-based volumetric reconstructions [21, 22]. However, the absence of CT scanners in many Brazilian Forensic Institutes imposes the need for adaptation and creative solutions. The 3D–2D methodology, which consists of superimposing a three-dimensional model (obtained from an original antemortem CT) onto a two-dimensional image (such as a postmortem radiograph), emerges as a highly effective and low-cost solution.

One of the main advantages of this technique is the ability to generate a 3D reference model from AM data – as among which are volumetric reconstruction and segmentation of the frontal sinus from an AM CT scan - and virtually superimpose it onto a PM 2D image. This process, performed entirely using open-source software, requires no licensing costs, making it accessible to institutions with restricted budgets. Moreover, the flexibility to rotate and rescale the 3D model in real time within a virtual environment minimizes visual inconsistencies inherent in purely 2D–2D superimposition. In 2D–2D comparisons, minimal positioning variations can lead to inconsistencies or require multiple radiographic acquisitions through trial and error to “reproduce” the original image. With 3D–2D superimposition, real-time adjustment of the 3D model ensures accurate comparison even when PM examinations are less precise [13].

This technique not only optimizes the identification process but also serves as a powerful incentive for less-equipped Forensic Institutes. By demonstrating that effective and efficient results can be achieved without massive investment in CT equipment, the emphasis shifts toward workforce training. Expertise in 3D technology and forensic imaging thus becomes the most strategic asset, enabling professionals to maximize available resources, for instance volumetric reconstruction and segmentation of anatomical structures from original AM CT scans, even when the Forensic Institute does not have its own CT scanner for PM examinations. Therefore, 3D–2D methodology is not only a viable alternative but also a pathway to democratizing access to advanced forensic identification techniques in Brazil, promoting excellence and justice at all levels [29–31, 34].

The incorporation of 3D technology into forensic anthropology represents a significant advance in forensic science. The use of the frontal sinus as a comparative structure in 3D–2D modeling illustrates the potential of digital techniques for identifying individuals who cannot be identified by traditional methods. Furthermore, this methodology can be extended to other anatomical regions, such as the sphenoid sinus, palate, and dental structures, contributing to the consolidation of more objective, precise, and reproducible methods [21, 26, 27]. Although it remains a challenge, including protocol standardization and the interpretation of damaged images, continuous progress in artificial intelligence and computational modeling points to a promising future for human identification based on three-dimensional imaging [26, 27].

This case report highlights the importance of forensic anthropologists and forensic odontologists in human identification, particularly in cases involving frontal sinus analysis through CT imaging combined with 3D reconstruction. The use of 3D–2D superimposition technique proved to be a highly effective tool, enabling detailed morphological comparison between AM and PM examinations. The accuracy observed in this process demonstrates the potential of this methodology in obtaining objective, reliable, and reproducible results, even in complex contexts such as advanced decomposition. Thus, the combination of expertise in 3D technology, anatomical knowledge, forensic imaging, and quantitative methods reaffirms the value of forensic dentistry as an essential pillar of human identification.

## Supplementary Information

Below is the link to the electronic supplementary material.

Supplementary Material 1

## Declarations

### Submission declaration

This manuscript has not been published and is not under consideration for publication elsewhere.
