# Supplementary material for: 3D modeling of the frontal sinus as a contribution tool for human identification - a case report
Source: Int J Legal Med. 2026 Apr 27;140(5):3011–7. doi: 10.1007/s00414-026-03815-z (PMC13499824; doi:10.1007/s00414-026-03815-z)
Supplement: Supplementary file 1 [file 414_2026_3815_MOESM1_ESM.docx]

**3D MODELING OF THE FRONTAL SINUS AS A CONTRIBUTION TOOL FOR HUMAN IDENTIFICATION - A CASE REPORT**

Sandra Gabolli^1^

Ana Clara Oliveira^2^

Paulo Miamoto^3^

Maria Isabel Villalobos^4^

^1^Graduate Student, Department of Medicine, Pontifícia Universidade Católica de Minas Gerais, Betim, Brazil <https://orcid.org/0009-0000-2904-1090>

^2^Graduate Student, Department of Dentistry, Pontifícia Universidade Católica de Minas Gerais, Belo Horizonte, Brazil https://orcid.org/0009-0008-3587-2200

^3^Forensic Odontology and Anthropology Section, Scientific Police of Santa Catarina, Florianópolis, Brazil https://orcid.org/0000-0003-0247-9433

^4^Post-Graduate Program in Dentistry, Pontifícia Universidade Católica de Minas Gerais, Belo Horizonte, Brazil <https://orcid.org/0000-0002-8675-9186>

**Correspondence address:**

Maria Isabel Villalobos – [contato@misabel.com.br](mailto:contato@misabel.com.br)

Graduate Program in Dentistry, Pontifícia Universidade Católica de Minas Gerais, Av. Dom José Gaspar 500, Prédio 46, Sala110, CEP 30535-901, Belo Horizonte, Minas Gerais, Brazil

**Acknowledgement**

The authors would like to thank Santa Catarina Scientific Police that supported this work. This study was financed in part by the Coordenação de Aperfeiçoamento de Pessoal de Nível Superior - Brasil (CAPES) Finance Code 001.
